# Supplementary material for: Random survival forest model for early prediction of Alzheimer’s disease conversion in early and late Mild cognitive impairment stages
Source: PLoS One. 2024 Dec 13;19(12):e0314725. doi: 10.1371/journal.pone.0314725 (PMC11642905; doi:10.1371/journal.pone.0314725)
Supplement: Attachment — Submitted filename: Response to Reviewers.docx [file pone.0314725.s002.docx]

**Manuscript Number**: PONE-D-24-27193

**Original Article Title: “**Random Survival Forest Model for Early Prediction of Alzheimer's Disease Conversion in Early and Late Mild Cognitive Impairment Stages”

**To:** PLOS ONE Editor

**Re:** Response to reviewers

Dear Editor,

Thank you for allowing a re-submission of our manuscript, with an opportunity to address the reviewers’ comments.

We are uploading (a) Our point-by-point response to the comments (below) (response to reviewers), (b) An updated manuscript with red highlighting indicating changes, (c) A clean updated manuscript without highlights, (d) An updated cover letter, and (e) All updated figures.

Yours Sincerely,
Dr. Asim Waris
Associate Professor,
School of Mechanical and Manufacturing Engineering,
National University of Sciences & Technology (NUST), Islamabad, Pakistan
Tel.: +92(0) 5190856068
E-mail: [asim.waris@smme.nust.edu.pk](mailto:asim.waris@smme.nust.edu.pk)

**Response to Reviewer 1**

**Concern #1:** The manuscript closely resembles the work by Sarica et al., with the only apparent novelty being the comparison between eMCI and lMCI. More elaboration on how this work advances the field beyond previous studies is necessary.

**Author Response:** Thank you for your thoughtful feedback. We acknowledge that our work closely resembles the study by Sarica et al. and appreciate the opportunity to clarify how we have built upon and advanced it. Our contributions are as follows:

- While our research builds on the foundation of Sarica et al., we have focused on an important aspect: analyzing how the progression to Alzheimer's Disease (AD) differs between early (eMCI) and late (lMCI) stages of Mild Cognitive Impairment. This detailed comparison has not been extensively covered in previous work, and we believe it adds significant value to the field.
- To enhance precision and accuracy, we built separate models for eMCI and lMCI stages. This approach allows for a more tailored analysis of disease progression, rather than treating MCI as a uniform stage.
- We compared six machine learning models to identify the most effective in predicting AD conversion, offering a broader evaluation than previous studies. This comparative approach highlights the strengths of different models in these distinct MCI stages.
- For the eMCI dataset, which faced class imbalance, we implemented a robust strategy to handle the imbalance. This approach ensures that our model produces reliable results without overfitting or introducing bias, a common risk when dealing with imbalanced data.
- Our study also performed a multimodal comparison, evaluating neuropsychological assessments, neuroimaging (MRI + PET), and CSF biomarkers both individually and in combination. This comprehensive analysis helped determine whether combining modalities improves predictive performance or if a single modality can perform equally well.
- A key advancement in our work is generating individual survival curves from baseline data. These curves provide personalized risk assessments for each patient, offering valuable information to clinicians in both eMCI and lMCI stages.

**Author Action:** Thank you for your valuable comments. In response, we have made additions to both the Introduction and Discussion sections to emphasize the novel aspects of our study and how it builds upon the work of Sarica et al. These additions highlight the unique contributions of our research, particularly the detailed comparison of early and late MCI stages, handling imbalance in dataset and the generation of personalized survival curves.

- *We have added the necessary revisions in the Introduction section, from page 5, line 111, to page 6 line 124:*

‘While these studies have made substantial contributions, there is still a lack of extensive research on predicting time-to-AD conversion in the eMCI and lMCI stages using multimodal data. According to studies, the rate of AD progression differs among stages [21].  Hence it is crucial to develop ML models specific to each MCI stage, so they can capture the distinct patterns of each stage to generate more precise and personalized predictions. Such a stage-specific approach can enable clinicians to identify the individual at risk, allowing for timely interventions and better patient outcomes [21]. By developing separate models for eMCI and lMCI, our study addresses this need, advancing the precision of diagnosis and prognosis across different points in the disease continuum. Additionally, the handling of data imbalance in survival analysis datasets is a relatively underexplored challenge, especially in AD progression. To bridge these gaps, we implemented a comprehensive strategy using multiple ML survival models to predict AD conversion risks in eMCI and lMCI patients separately. This approach not only provides the first stage-specific ML-based survival analysis for MCI but also introduces methods to mitigate data imbalance, enabling more reliable and personalized risk assessments for clinical decision-making.’

- *We have added the necessary revisions in the Discussion section, from page 26, line 538, to page 27, line 553:*

‘Our results align with previous research, which has demonstrated the effectiveness of RSF in predicting time-to-event scenarios in both clinical and research settings [28], [29] [35], [36]. RSF possesses several key features that make it a reliable approach for disease forecasting, including built-in mechanisms to reduce overfitting, effectively handling high dimensional data, capturing complex relationships between predictors and survival outcomes and absence of convergence issues [37], [38]. These attributes contribute to its effectiveness in medical research and clinical applications. Our study builds upon the previous work by developing separate models for early and late MCI stages and identifying the key features that serve as important predictors for each stage. This approach enables more accurate predictions tailored to the unique progression patterns of each group. We also addressed the challenge of dataset imbalance by using oversampling techniques, leading to more reliable and unbiased model performance. We generated individual survival curves for both progressive and stable subjects in both datasets. The models used to generate these curves were trained separately on the eMCI and lMCI data. This approach enhances the precision and accuracy of the survival curves and is better suited to the specific characteristics of each stage. These results can help clinicians make early, personalized predictions of Alzheimer's progression, enabling timely interventions and better resource planning for at-risk patients.’

**Concern #2:** The high c-index for eMCI is surprising, given the low number of conversions to AD in this cohort. This raises concerns about the robustness of the findings.

**Author Response:** Thank you for raising this important concern regarding the unexpectedly high c-index for the eMCI cohort. Upon reviewing this comment, we realized that our original findings were not as robust as initially believed. The main reason behind this was the significant class imbalance in the eMCI dataset, which we had attempted to address by balancing the entire dataset before splitting it into training and test sets. As you correctly pointed out, this led to data leakage, where some oversampled data points appeared in both the training and test sets, resulting in higher accuracy and a biased model. Consequently, the test set was not truly "unseen" during evaluation, which compromised the reliability of our results.

To rectify this, we adjusted our methodology. Instead of oversampling the entire dataset before splitting, we first divided the dataset into training and test sets and then oversampled the minority class (uncensored subjects) only in the training set. This way, the test set remained unbalanced, representing real-world data distribution, and contained unseen data for proper evaluation. (More on this in the response to Concern#4)

Additionally, we incorporated cross-validation and hyperparameter optimization to further ensure the model's robustness. With these changes, the new results are reliable, achieving a c-index of 0.90 and IBS of 0.10 for the Random Survival Forest model, which we believe reflects the true predictive power of the model.

**Author Action:** We have revised the 'Target Imbalance' section in the Methodology and made additions to the Results and Discussion sections. These updates explain how we address target imbalance to ensure robust findings and include updated results for the eMCI dataset.

- *We have revised the Abstract, on page 1, line 32 to line 33:*

‘For eMCI, RSF trained on multimodal data achieved a C-Index of 0.90 and an IBS of 0.10. For lMCI, the C-Index was 0.82 and the IBS was 0.16.’

- *We have revised the section ‘Target Imbalance’ in the Methodology, from page 10, line 208, to line 219, and updated the Figures 3 and 4:*

*2.3.4 Target Imbalance:*

‘For prediction labels, patients were categorized into two groups: those showing progression of the disease (labeled '1') and those who did not (labeled '0'). Figure 3 compares the distribution of censored (stable) and uncensored (AD converters) subjects in both the eMCI and lMCI datasets. A noticeable imbalance is present in the eMCI group, where there are significantly fewer uncensored cases, whereas the lMCI group shows a more balanced distribution. Imbalanced datasets can lead to biased models that do not perform well on new data [22]. To address this, we used the 'random oversampler' from the sklearn library, which balances the class distribution by randomly duplicating samples from the minority class. We first split the dataset into training and testing sets, then oversampled the minority class in the training set to balance it before model training, which is illustrated in Figure 4. The testing set was left unbalanced to reflect real-world conditions and ensure the model was evaluated on unseen, naturally distributed data.’

**Figure 3.** Distribution of censored and uncensored data in eMCI and lMCI groups.

**Figure 4.** Balancing the training set of eMCI group.

- *We have updated the Results section, on page 17, line 352 to line 357, and Figure 6.*

‘This section discusses the performance of ML models trained on multimodal data that includes all features. As compared to other models, RSF had the highest accuracy on both datasets. All the ML models outperformed the traditional CoxPH model in both datasets. For eMCI group, among the ensemble-based models, RSF showed the best performance (C-Index= 0.90 ± 0.03, IBS= 0.10 ± 0.02), followed by ) XST (C-Index= 0.86 ± 0.02, IBS= 0.10 ± 0.03) and Gradient Boosting (C-Index= 0.82 ± 0.02, IBS= 0.10 ± 0.03).’

**Figure 6.** Heatmap showing performance of models measured by C-Index and IBS for early and late MCI.

- *We have updated the Results section on page 19, line 382 – 385:*

‘For the eMCI group, RSF trained on cognitive features achieved a C-Index of 0.85 ± 0.02 and an IBS of 0.11 ± 0.02 compared to Imaging features (C-Index= 0.76 ± 0.02, IBS= 0.14 ± 0.02, p<0.05) and CSF biomarkers (C-Index= 0.74 ± 0.03, IBS= 0.16 ± 0.02, p<0.05).’

- *Furthermore, we have updated Figure 8 which illustrates the individual survival curves for individuals with eMCI and lMCI. The balancing approach has resulted in enhanced survival curves:*

|    (a) **Progressive eMCI subject** |    (b) **Non-Progressive eMCI subject** |
| --- | --- |

**Figure 8.** Predicted survival estimates for subjects with progressive eMCI and lMCI as well as those with non-progressive eMCI and lMCI. The red line refers to the actual event times for progressive/uncensored patients and the actual censoring time for non-progressive/censored patients.

- *We have made additions to the Discussion section, on page 24, line 490 to 510:*

‘Handling missing data and target imbalance is a common challenge in biomedical research. To address this, we used KNN imputation to manage missing data. In the eMCI dataset, there was a significant imbalance between the number of subjects who progressed to AD (uncensored) and those who remained stable (censored). Imbalanced datasets can lead to biased models and overfitting, which affects the reliability of results [33]. Table 4 shows when the model was trained on the imbalanced dataset, it achieved 98% accuracy on the training set but only 71% on the test set. This indicates overfitting, suggesting that the model may have learned patterns specific to the training data that do not generalize to new data. Datta et al. [22] achieved improved predictive performance of their Cox elastic net regression models when trained on sampled data. This highlights the effectiveness of integrating sampling methods with survival analysis techniques to improve predictive performance in biomedical research. Oversampling is one of several sampling techniques used to address target imbalance in disease prediction studies [34]. To address imbalance in eMCI dataset, we first split the data into training and test sets. The training set was then balanced by oversampling the minority class (subjects progressing to AD), while the test set remained unbalanced so that the final performance evaluation remains unbiased. After applying this balancing strategy, the model’s performance improved significantly, achieving 97% accuracy on the training set and 90% on the test set. This improvement suggests that balancing the training data enhanced the model’s generalizability and reliability. Balancing the entire dataset before the train-test split was avoided, as it could cause data leakage, with some data points appearing in both the training and test sets, leading to overfitting and unreliable model evaluation. After completing preprocessing, we proceeded with training and evaluating the models.’

**Table 4.** RSF's performance before and after applying data balancing.

| **Model Setup** | **C-Index - Training set** | **C-Index – Test set** |
| --- | --- | --- |
| RSF trained on imbalanced dataset | 0.98 | 0.71 |
| RSF trained on balanced training set | 0.97 | 0.90 |

**Concern #3:** The authors have not reported the number of events or censored data per year, which is crucial information.

**Author Response:** Thank you for bringing this to our attention. We agree that reporting the number of events and censored data per year is crucial for understanding the dataset's characteristics. We have now included this information in the manuscript as a figure, illustrating the number of censored events and total events per year for each dataset (eMCI and lMCI).

**Author Action:** Figure 2 has been added on page 8, line 172, providing details on the number of events and censored data for both eMCI and lMCI cohorts by year.

**Figure 2.** Censored and uncensored data distribution per year in Early and late MCI individuals.

**Concern #4:** The technique used for group balancing was not declared. It is important to note that balancing survival data is generally not recommended in the literature.

**Author Response:** Thank you for raising this important concern regarding group balancing in survival analysis. In response, we acknowledge that balancing survival data is not always recommended in the literature due to the potential risks of introducing bias or overfitting. However, in our study, the decision to balance the training data was carefully considered due to the severe imbalance in the eMCI dataset, where the minority class (subjects progressing to AD) was significantly underrepresented. This imbalance posed a risk of skewing the model towards the majority class, which could lead to poor performance in predicting progression to AD, the key outcome of our study.

We used sklearn's oversampling technique to balance the training set only, ensuring the test set remained imbalanced to avoid data leakage and overfitting. This strategy allowed the model to learn effectively from the minority class, improving test set accuracy from 71% to 90%, while maintaining high training accuracy (97%). In contrast, without balancing, the model showed substantial overfitting (98% training accuracy vs. 71% test accuracy). The results validated our approach, as balancing the training set significantly improved the model’s generalizability.

The balancing approach also improved the generation of individual survival curves by ensuring the model learned from both stable and progressive patients, leading to more accurate risk assessments. This is crucial for high-risk patients, who were underrepresented in the original dataset.

We fully agree that balancing survival data can be problematic if not done carefully, but in our case, the combination of balancing only the training set and keeping the test set unbalanced allowed us to mitigate overfitting and improve both predictive performance and the accuracy of individual survival curves. Few studies have demonstrated the effectiveness of combining sampling methods with survival analysis techniques to enhance predictive performance in biomedical research. One notable study by Datta et al. involved training Cox elastic net regression models on both upsampled and downsampled data, leading to improved performance compared to models trained on imbalanced datasets [1]. Consequently, we believe that this balanced approach was essential and effective, given the significant imbalance in our dataset, enabling us to achieve more reliable and clinically meaningful outcomes.

We hope this clarifies our rationale and reassures you that the balancing strategy was applied with caution and justified by the challenges posed by the data.

**Author Action:** We have revised the target imbalance section to clarify the balancing strategy. Additionally, we have updated the results, discussion section, and all corresponding figures

- *We have revised Target Imbalance section in Methodology, on page 10, from line 208 to line 219, to provide a more detailed explanation of the upsampling technique used and our rationale for its application. We have also updated the corresponding Figures 3 and 4, which have been shared in response to Concern No. 2*:

‘ *2.3.4 Target Imbalance:*

For prediction labels, patients were categorized into two groups: those showing progression of the disease (labeled '1') and those who did not (labeled '0'). Figure 3 compares the distribution of censored (stable) and uncensored (AD converters) subjects in both the eMCI and lMCI datasets. A noticeable imbalance is present in the eMCI group, where there are significantly fewer uncensored cases, whereas the lMCI group shows a more balanced distribution. Imbalanced datasets can lead to biased models that do not perform well on new data [22]. To address this, we used the 'random oversampler' from the sklearn library, which balances the class distribution by randomly duplicating samples from the minority class. We first split the dataset into training and testing sets, then oversampled the minority class in the training set to balance it before model training, which is illustrated in Figure 4. The testing set was left unbalanced to reflect real-world conditions and ensure the model was evaluated on unseen, naturally distributed data.’

- *We have revised the discussion and explained the balancing strategy used in our study, on page 24, line 3-23:*

‘Handling missing data and target imbalance is a common challenge in biomedical research. To address this, we used KNN imputation to manage missing data. In the eMCI dataset, there was a significant imbalance between the number of subjects who progressed to AD (uncensored) and those who remained stable (censored). Imbalanced datasets can lead to biased models and overfitting, which affects the reliability of results [33]. Table 4 shows when the model was trained on the imbalanced dataset, it achieved 98% accuracy on the training set but only 71% on the test set. This indicates overfitting, suggesting that the model may have learned patterns specific to the training data that do not generalize to new data. Datta et al. [22] achieved improved predictive performance of their Cox elastic net regression models when trained on sampled data. This highlights the effectiveness of integrating sampling methods with survival analysis techniques to improve predictive performance in biomedical research. Oversampling is one of several sampling techniques used to address target imbalance in disease prediction studies [34]. To address imbalance in eMCI dataset, we first split the data into training and test sets. The training set was then balanced by oversampling the minority class (subjects progressing to AD), while the test set remained unbalanced so that the final performance evaluation remains unbiased. After applying this balancing strategy, the model’s performance improved significantly, achieving 97% accuracy on the training set and 90% on the test set. This improvement suggests that balancing the training data enhanced the model’s generalizability and reliability. Balancing the entire dataset before the train-test split was avoided, as it could cause data leakage, with some data points appearing in both the training and test sets, leading to overfitting and unreliable model evaluation. After completing preprocessing, we proceeded with training and evaluating the models.’

**Table 4.** RSF's performance before and after applying data balancing.

| **Model Setup** | **C-Index - Training set** | **C-Index – Test set** |
| --- | --- | --- |
| RSF trained on imbalanced dataset | 0.98 | 0.71 |
| RSF trained on balanced training set | 0.97 | 0.90 |

**Concern #5:** It is unclear why the authors chose to use years instead of months for the timing of events.

**Author Response:** Thank you for your thoughtful feedback regarding the choice of using years instead of months for the timing of events. We chose to use years for the following reasons:

- Our approach aligns with the methodologies used in several key studies on Alzheimer's Disease progression, ensuring consistency with the existing body of literature. [2], [3]. [4], [5].
- Clinically, significant changes in the progression from MCI to AD are typically observed over longer periods, making yearly intervals more relevant for understanding the natural history of the disease and predicting patient outcomes.
- Finally, analyzing the data on a yearly basis allowed for more stable model performance, ensuring a sufficient number of events in each time period, which is crucial for the robustness of survival analysis models.

We hope this explanation clarifies our choice and addresses your concerns.

**Author Action:** None.

**Concern #6:** Table 1 should report the demographics of eMCI and lMCI cohorts, split between those who converted to AD and those who did not, along with the occurrence of events/censorship.

**Author Response:** Thank you for your feedback. We appreciate this suggestion and agree that including this information would provide a clearer understanding of the cohort characteristics.

**Author Action:**

- *We have updated Table 2, to include the demographics of eMCI and lMCI cohorts, split between converters and non-converters, and the occurrence of events/censorship. We also conducted a statistical comparison of the features between eMCI and lMCI groups to identify any significant differences between the two stages.*

**Table 2.** Data statistics of eMCI and lMCI groups in this study.

|  | | eMCI | | lMCI | |  | | eMCI & lMCI |
| --- | --- | --- | --- | --- | --- | --- | --- | --- |
| **Features** | **Progressive (23)** | **Stable (268)** | ***p*-value^a^** | **Progressive (245)** | **Stable (301)** | ***p*-value^a^** | ***p*-value^*^** | |
| **Female, gender (n%)** | 8 (34.7%) | 119 (44.4%) | 0.37 | 97 (40%) | 119 (39.5%) | 0.74 | 0.17 | |
| **Male, gender (n%)** | 15 (65.3%) | 149 (55.6%) | 0.37 | 148 (60%) | 186 (61.7%) | 0.74 | 0.17 | |
| **Age** | 72.5 ± 6.25 | 71.4 ± 7.65 | 0.49 | 74 ± 7.2 | 73.9 ±7.7 | 0.9 | **< 0.001** | |
| **Education** | 15.65 ± 2.60 | 15.89 ± 2.68 | 0.68 | 15.9 ± 2.7 | 15.7 ± 3.1 | 0.55 | 0.95 | |
| **CDRSB** | 2.13 ± 0.94 | 1.24 ± 0.69 | **< 0.001** | 1.9 ± 0.9 | 1.47 ± 0.8 | **< 0.001** | **< 0.001** | |
| **ADAS13** | 15.6 ± 5.63 | 12.6 ±5.3 | **0.01** | 21.3 ± 5.9 | 16.8 ± 6.1 | **< 0.001** | **< 0.001** | |
| **ADAS11** | 9.7 ± 4.1 | 7.89 ± 3.4 | **0.01** | 13.2 ± 4.3 | 10.3 ± 4.2 | **< 0.001** | **< 0.001** | |
| **MMSE** | 28.3 ± 1.6 | 28.2 ± 1.5 | 0.8 | 26.7 ± 1.7 | 27.5 ± 1.79 | **< 0.001** | **< 0.001** | |
| **RAVLT.immediate** | 33.1 ± 9.3 | 39.7 ± 10.5 | **0.004** | 28 ± 7.5 | 33.3 ± 9.8 | **< 0.001** | **< 0.001** | |
| **RAVLT.learning** | 4.5 ± 2.7 | 5.27 ± 2.4 | 0.15 | 2.73 ± 2.13 | 3.8 ± 2.4 | **< 0.001** | **< 0.001** | |
| **RAVLT.forgetting** | 4.5 ± 2.6 | 4.28 ± 2.6 | 0.68 | 4.85 ± 2.1 | 4.79 ± 2.4 | 0.7 | ***<* 0.001** | |
| **RAVLT.perc.forgetting** | 57.4 ±35 | 46.8 ± 30 | 0.11 | 76.5 ± 28 | 62 ± 31 | **< 0.001** | **< 0.001** | |
| **FAQ** | 5.56 ± 5.1 | 1.7 ± 2.7 | **< 0.001** | 5.34 ± 4.8 | 2.7 ± 3.8 | **< 0.001** | **< 0.001** | |
| **FDG-PET** | 5.9 ± 0.6 | 6.4 ± 0.58 | **< 0.001** | 5.8 ± 0.6 | 6.17 ± 0.6 | **< 0.001** | **< 0.001** | |
| **Ventricles, × 10^3^** | 40. 5 ± 19.8 | 34.9 ± 20.7 | 0.23 | 43.9 ± 22.9 | 42.4 ± 25.2 | 0.5 | **< 0.001** | |
| **Hippocampus, × 10^3^** | 6.7 ±1.0 | 7.2 ± 1.0 | **0.02** | 6.1 ± 1.0 | 6.7 ± 1.0 | **< 0.001** | **< 0.001** | |
| **Whole Brain, × 10^5^** | 10.6 ± 1.04 | 10.7 ± 1.06 | 0.66 | 9.97 ± 1.1 | 10.2 ± 1.0 | **0.003** | **< 0.001** | |
| **Entorhinal, × 10^3^** | 3.6 ± 0.9 | 3.7 ± 0.6 | 0.27 | 3.08 ± 0.7 | 3.5 ± 0.7 | **< 0.001** | **< 0.001** | |
| **Fusiform, × 10^3^** | 1.8 ± 2.8 | 1.8 ± 2.6 | 0.2 | 16.08 ± 2.4 | 17.39 ± 2.5 | **< 0.001** | **< 0.001** | |
| **Mid Temporal, × 10^3^** | 1.9 ± 2.7 | 20.8 ± 2.6 | **0.005** | 17.9 ± 2.8 | 19.7 ± 2.7 | **< 0.001** | **< 0.001** | |
| **ABETA** | 768.5 ± 411 | 1126 ± 442 | **< 0.001** | 716 ± 317 | 941 ± 435 | **< 0.001** | **< 0.01** | |
| **TAU** | 286 ± 179 | 256 ± 118 | 0.27 | 345 ± 120 | 290 ± 151 | **< 0.001** | **< 0.001** | |
| **PTAU** | 28.7 ± 21.2 | 24.2 ± 13.1 | 0.14 | 34 ± 13.6 | 28.3 ± 16.5 | **< 0.001** | **< 0.001** | |

**Significant results indicated in bold, with *p* < 0.05.**

**^a^ Statistical comparison between progressive and stable individuals in the eMCI & lMCI groups respectively.**

***Statistical comparison between features of eMCI & lMCI groups.**

- *We have also updated the Results section, on page 15, line 6 to line 9:*

‘The statistical results in Table 2 show statistically significant differences between almost all features of eMCI and lMCI datasets. Most features showed statistically significant differences between progressive lMCI and stable lMCI. Similarly, most cognitive tests revealed statistically significant differences between progressive eMCI and stable eMCI.’

**Concern #7:** The percentage of missing data per diagnosis is not provided, which is fundamental in determining whether imputation methods are appropriate.

**Author Response:** Thank you for your valuable suggestion. We agree that the percentage of missing data is crucial for transparency and for evaluating the suitability of imputation methods.

**Author Action:**

- *We have added a new table (Table 1) on page 9, line 5 in the Methods detailing the percentage of missing data per feature, and updated the Imputation part in Methodology section, on page 8, line 18 to 20:*

‘Having missing values is a problem that is often encountered in medical studies. Similarly, there were missing values in a few features in our datasets, which can be observed in Table 1. Approximately 7.71% of the data was missing from the entire dataset. Deleting entire rows with missing values results in the loss of important information.’

**Table 1.** Number of missing values in features of eMCI and lMCI datasets.

|  | **Missing Values** | |
| --- | --- | --- |
| **Features** | **eMCI (291)** | **lMCI (546)** |
| ABETA | 32 | 213 |
| ADAS11 | 3 | 1 |
| ADAS13 | 3 | 4 |
| Entorhinal | 34 | 92 |
| FAQ | 3 | 4 |
| FDG | 5 | 197 |
| Fusiform | 34 | 92 |
| Hippocampus | 34 | 92 |
| MidTemp | 34 | 92 |
| PTAU | 32 | 213 |
| RAVLT.forgetting | 1 | 1 |
| RAVLT.immediate | 1 | 1 |
| RAVLT.learning | 1 | 1 |
| RAVLT.perc.forgetting | 1 | 2 |
| TAU | 32 | 213 |
| Ventricles | 34 | 92 |
| WholeBrain | 34 | 92 |

**Concern #8:** The Results section should be separated from the Discussion to avoid confusion.

**Author Response:** Thank you for this suggestion. We agree that separating these sections would improve the manuscript's clarity.

**Author Action:** We have revised the manuscript to separate the Results section from the Discussion.

- *We have separated the Results section, which starts from page 15, line 5 to page 22, line 17. The Discussion section starts from page 22, line 22 to page 27, line 4.*

**Concern #9:** In Figures 5 and 6, the feature rankings should be ordered by importance to clearly highlight the most significant variables.

**Author Response:** We agree with your point and understand the importance of clearly presenting the feature rankings. Ordering them by significance would certainly enhance the interpretability of the findings.

**Author Action:** Figures 5 and 6 are now Figure 7 (A) and (B). We have reordered the feature rankings in Figures 7 to display them in order of importance.

 **Figure 7.** Feature Importance results of eMCI and lMCI datasets. The error bars show the standard deviation, and each bar indicates the mean score.

**References:**

[1]	G. Datta, L. E. Alexander, M. A. Hinterberg, and Y. Hagar, “Balanced Event Prediction Through Sampled Survival Analysis,” *Systems Medicine*, vol. 2, no. 1, pp. 28–38, Dec. 2019, doi: 10.1089/sysm.2018.0015.

[2]	“Survival prediction models: an introduction to discrete-time modeling - PubMed.” Accessed: Oct. 07, 2024. [Online]. Available: https://pubmed.ncbi.nlm.nih.gov/35883032/

[3]	G. Mirabnahrazam *et al.*, “Predicting time-to-conversion for dementia of Alzheimer’s type using multi-modal deep survival analysis,” *Neurobiol Aging*, vol. 121, pp. 139–156, Jan. 2023, doi: 10.1016/j.neurobiolaging.2022.10.005.

[4]	R. Brookmeyer, M. M. Corrada, F. C. Curriero, and C. Kawas, “Survival Following a Diagnosis of Alzheimer Disease,” *Archives of Neurology*, vol. 59, no. 11, pp. 1764–1767, Nov. 2002, doi: 10.1001/archneur.59.11.1764.

[5]	M. L. Haaksma *et al.*, “Survival time tool to guide care planning in people with dementia,” *Neurology*, vol. 94, no. 5, pp. e538–e548, Feb. 2020, doi: 10.1212/WNL.0000000000008745.

**Response to Reviewer 2**

**Comment#1:** I find this a thoroughly carried out study, which has the potential to advance the field. The paper is well-written. Thus, I recommend the manuscript for publication with the suggestion that the code reproducing the key results is made available as supplementary information with the paper.

**Author Response:** Thank you for your thorough and positive feedback on our manuscript. We truly appreciate your recognition of our work's potential to advance the field and your thoughtful suggestions. In response to your recommendation, we would like to assure you that the code reproducing the key results will be made available at the time of publication, ensuring full transparency and reproducibility of our findings.

______________________________________________________________________________
